# Establishment and characterization of a *CCND1*-rearranged non-mantle cell lymphoma cell line and patient-derived xenograft model

**DOI:** 10.1038/s41375-025-02849-3

**Published:** 2026-01-26

**Authors:** Claus-Moritz Gräf, Moritz Reese, Angela Vicente-Luque, Nicolas Mönig, Charlotte Bruzeau, Ferran Nadeu, Maria Latacz, Johanna Bihler, Jörn Meinel, Maria Cartolano, Martin Peifer, Sílvia Beà, Elias Campo, Melanie Thelen, Paul J. Bröckelmann, Ron D. Jachimowicz

**Affiliations:** 1 Max Planck Institute for Biology of Ageing, Cologne, Germany; 2 Department I of Internal Medicine, Center for Integrated Oncology Aachen Bonn Cologne Duesseldorf (CIO ABCD), Faculty of Medicine and University Hospital Cologne, University of Cologne, Cologne, Germany; 3 Institut d’Investigacions Biomèdiques August Pi i Sunyer (IDIBAPS), Barcelona, Spain; 4 Centro de Investigació n Biomédica en Red de Cá ncer (CIBERONC), Madrid, Spain; 5 Department of Pathology, University Hospital of Cologne, Cologne, Germany; 6 Department of Translational Genomics, Faculty of Medicine and University Hospital Cologne, University of Cologne, Cologne, Germany; 7 Centre for Molecular Medicine, University of Cologne, Cologne, Germany; 8 Hospital Clínic de Barcelona, Barcelona, Spain; 9 Universitat de Barcelona, Barcelona, Spain; 10 Cologne Excellence Cluster on Cellular Stress Response in Aging-Associated Diseases, University of Cologne, Cologne, Germany

**Keywords:** Cancer models, Cancer genomics, B-cell lymphoma, Cancer therapeutic resistance

## To the Editor:

Accurate diagnosis of distinct lymphoma subtypes critically informs contemporary therapeutic strategies, which increasingly incorporate targeted agents to exploit disease-specific molecular vulnerabilities [1, 2]. Nevertheless, diagnostic challenges remain, and discordance rates of up to 60% between local and reference pathologists were recently reported in a study of >31,000 patients [3]. Besides integrated clinical and phenotypic characterization, precise diagnosis often requires additional molecular and genetic analyses.

Cyclin D1 overexpression due to *CCND1* rearrangement (*CCND1*-R) involving immunoglobulin (IG) genes is considered a hallmark of mantle cell lymphoma (MCL). Recently, however, Cyclin D1 overexpression due to *CCND1*-R has also been identified in other B-cell lymphomas [4–6]. Moreover, the morphological and phenotypic spectrum of MCL ranges from small-sized to large pleomorphic and blastoid cells, and negativity for CD5 and SOX11. In light of varying treatment strategies, distinguishing these cases accurately from other lymphomas poses an important diagnostic challenge.

Recently, unusual IGH class-switch recombination (CSR) or somatic hypermutation (SHM) was reported as an underlying mechanism for *CCND1*-R in such non-MCL lymphomas [7]. To our knowledge, in vitro or in vivo model systems of these lymphomas are lacking (Fig. S1A, Supplementary Methods). We report the establishment and comprehensive characterization of the *CCND1*-R non-MCL cell line *HaJo* and the corresponding patient-derived xenograft (PDX) model to close this gap and facilitate biological characterization and preclinical testing of novel therapies.

**Fig. 1:**
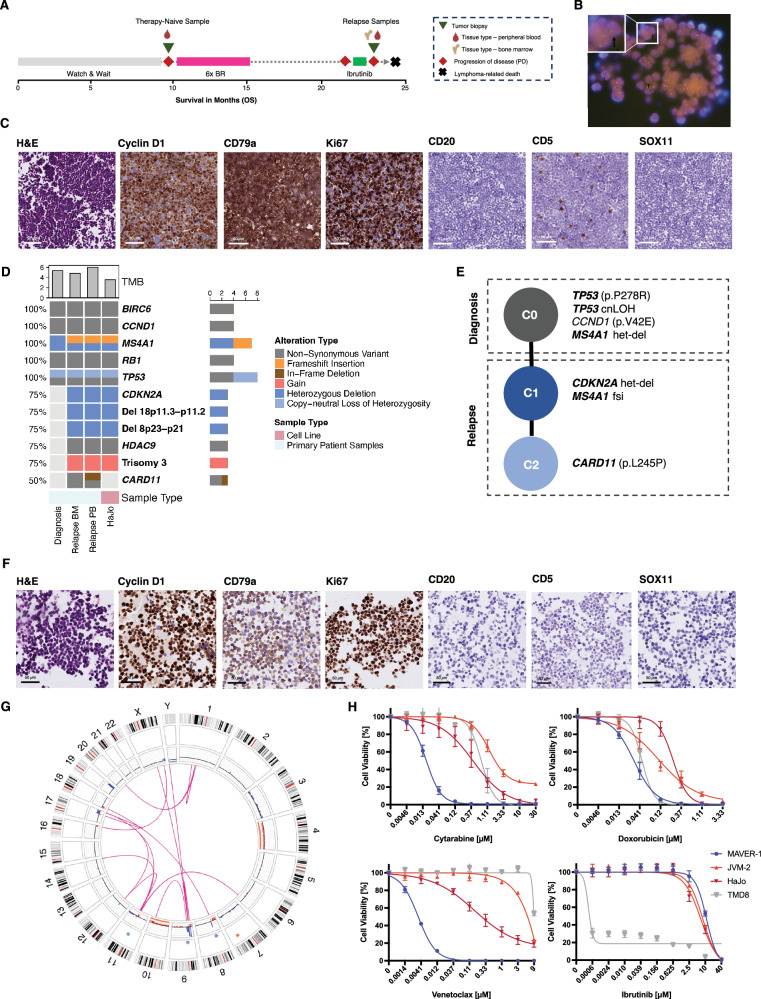
Establishment and molecular characterization of the CCND1-R non-MCL cell line HaJo. **A** The treatment-naive sample (green triangle) was collected from a 74-year-old male patient after disease progression (red diamond) under a watch-and-wait strategy. First-line therapy with six cycles of bendamustine-rituximab (BR) resulted in partial remission lasting six months, followed by disease relapse. The patient was subsequently treated with ibrutinib but exhibited refractory disease. At this time, two additional tumor samples were collected from peripheral blood and bone marrow. The patient ultimately succumbed to his disease. **B** FISH using a *CCND1* break-apart probe on a section of a cell block derived from unsorted leukemic PBMCs. Arrows indicate nuclei with split signals consistent with a *CCND1* rearrangement (78/100 nuclei counted). **C** Immunohistochemistry (IHC) staining of cell blocks generated from the patient’s peripheral blood at ~×40 magnification, which was utilized to establish the cell line which we termed HaJo acknowledging the contributions of Hannah Goldfarb-Wittkopf and Johanna Bihler. Malignant cells are of smaller size, with nuclei that exhibit slight irregularities and occasional indentations, while the chromatin appears dispersed. **D** Oncoprint summarizing key genetic driver alterations identified in the patient samples and the HaJo cell line. The bar chart above displays the tumor mutational burden (TMB), calculated as the number of non-synonymous variants per megabase of covered exome. **E** Tumor phylogeny showing a linear pattern of clonal evolution across primary patient samples collected before and after treatment. **F** Representative IHC stainings of a cell block derived from the HaJo cell line at ~×40 magnification. **G** Circos plot showing genome-wide structural variants (pink lines) and copy number changes in the inner track identified by optical genome mapping. Gains are shown in red and losses in blue. **H** Relative cell viability of HaJo, the MCL cell lines MAVER-1 and JVM-2, as well as the ABC-DLBCL cell line TMD-8 serving as a positive control for ibrutinib sensitivity, assessed by CellTiter-Glo (CTG) after 72 hours of treatment with various concentrations of cytarabine, doxorubicin, venetoclax, and ibrutinib. Data represent the mean ± SD of at least three independent replicates.

HaJo was derived from leukemic peripheral blood of a 74-year-old male patient initially diagnosed with splenic marginal zone lymphoma (SMZL) and managed with a watch-and-wait approach outside our center (Fig. 1A, Table S1, Supplementary Methods). Upon disease progression, first-line treatment with six cycles of bendamustine and rituximab (BR) resulted in remission for six months. Subsequently, refractory disease with second-line ibrutinib treatment was observed and the patient was referred to our center. Here, the diagnosis was revised to MCL based on *CCND1*-R (Fig. 1B) with high Ki67 despite CD5- and SOX11-negativity (Fig. 1C). The patient rapidly succumbed to his disease without receiving further treatment. Due to the unusual disease course and immunophenotype, we comprehensively characterized all available tumor samples by whole-exome sequencing (WES), whole-transcriptome sequencing (WTS), B-cell receptor sequencing (BCRseq) and targeted panel sequencing of IG and CSR regions (Supplementary Methods).

Both therapy-naïve and relapsed lymphoma harbored a heterozygous deletion of *MS4A1* (Fig. 1D, E), as well as non-synonymous variants (NSV) in *BIRC6*, *CCND1*, *RB1*, and *TP53*, including a copy-neutral loss of heterozygosity (cnLOH) affecting *TP53* (Fig. 1D, E). At relapse following BR, the tumor acquired NSVs in *HDAC9* and *CARD11*, a heterozygous deletion of *CDKN2A*, and an additional frameshift insertion in *MS4A1*. Phylogenetic reconstruction revealed a linear clonal trajectory, with *TP53* as the founding driver (C0), followed by a dominant clone (C1) harboring the *CDKN2A* deletion and biallelic *MS4A1* inactivation (Fig. 1E). The latter explains the complete loss of CD20 expression, potentially in response to selective pressure from rituximab (Fig. 1A, C, F). A subclone (C2) that emerged during ibrutinib treatment carried a known gain-of-function *CARD11* mutation (p.L245P) (Fig. 1D, E, Fig. S1B) that was lost in the HaJo cell line, potentially due to absence of therapeutic pressure (Fig. 1D) [8]. At the copy number level, we identified trisomy 3 and deletions involving short arms of chromosome 8 and 18 upon relapse (Fig. 1D, Fig. S1,C), both consistent with the complex karyotype (Table S1, Table S3). Notably, structural abnormalities involving 9p13 cytoband include t(9;10)(p13;q24) and add(9)(p13), suggesting disruption of both alleles at the *CDKN2A* locus.

To further delineate the mechanism of *IG*::*CCDN1* rearrangement, present in both the treatment-naïve and refractory sample (Table S1), we performed targeted panel sequencing covering all IG V(D)J and CSR regions as previously described [7]. Despite this extensive panel, the rearrangement could not be detected by IgCaller (Table S2) [9]. Using optical genome mapping (OGM) we identified a complex pattern of structural and numerical alterations and two rearrangements between chromosomes 11 and 14. The breakpoints on chromosome 14 were downstream of IGHM and in the intergenic region between IGHJ and IGHD (Fig. 1G, Table S3, Supplementary Methods). This rules out the canonical RAG-mediated, V(D)J anomalous rearrangement seen in most SOX11 positive and negative MCL [9, 10]. In line with these findings, germline identity of IG heavy chain variable region gene (IGHV) was 91.7% (Table S2). Taken together, and in hindsight, the patient likely suffered from a transformation of a SMZL into a large B-cell lymphoma. The patient’s rapid and ultimately fatal disease course (Fig. 1A) underscores the critical unmet need for suitable model systems of *CCND1*-R non-MCL lymphomas.

To address this need, we first generated the HaJo cell line, mirroring the morphology (Fig. S1,D) and immunophenotype of the patient sample at relapse (Fig. 1C, F, Fig. S1,E-F). Additionally, the molecular landscape of the patient sample is largely conserved in HaJo, both in terms of genetic alterations (Fig. 1D) and transcriptional profiles (Fig. S1,G). Principal component (PC) analysis of transcriptome data revealed separation of SOX11-positive and SOX11-negative MCL cell lines along PC1, while HaJo clustered distinctly along PC2 (Fig. S1,H). Importantly, genes enriched in HaJo with therefore low loadings on PC2 are associated with marginal zone/memory B-cells (*CD24*, *FCRL1/5*), SHM activity (*AIM2*, *AICDA*), NF-κB signaling (*CARD11*, *BIRC3*, *NFATC1*, *CXCR5)*, and SMZL (*TNFAIP3*, *NOTCH3*, *SP140*, *FOXP1*), highly consistent with a transformed SMZL (Table S4) [11]. To explore suitability of HaJo for in vitro studies, we treated HaJo, as well as MCL (SOX11-positive: MAVER-1, SOX11-negative: JVM-2) and non-MCL (TMD-8) cell lines with increasing doses of cytarabine, doxorubicin, venetoclax, and ibrutinib (Fig. 1H). While HaJo displayed an intermediate sensitivity to chemotherapy, we did not observe sensitivity of HaJo towards ibrutinib, thereby mimicking the resistance of the patient to ibrutinib (Fig. 1H). Intriguingly, we identified overexpression of BCL2 in the patient (Fig. S1,I), and observed sensitivity to the BCL2 inhibitor venetoclax (Fig. 1H).

**Fig. 2:**
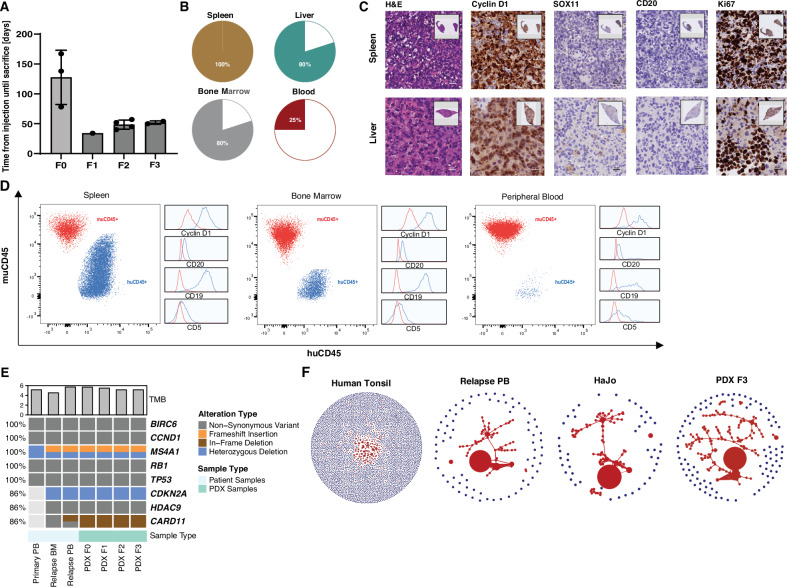
In vivo characterization of a CCND1-R non-MCL PDX model. **A** Bar plot depicting time from intravenous tumor cell injection to death across PDX passages in days. Each bar represents the mean ± SD. Individual data points correspond to single mice. **B** Relative fraction of organ infiltration as assessed by IHC staining. **C** Representative IHC images of spleen and liver from an F3 PDX mouse. H&E staining shows tumor cell infiltration in liver and spleen, with CCND1 and Ki67 overexpression, and SOX11 and CD20 negativity. **D** Flow cytometry plots showing murine CD45 and human CD45 expression, with additional depiction of expression of Cyclin D1, CD20, CD19, and CD5 in the corresponding populations by histograms in dissociated spleen tissue, bone marrow, and peripheral blood. **E** Oncoprint summarizing key genetic driver alterations identified in the primary patient sample and subsequent PDX passages. The bar chart above displays the tumor mutational burden (TMB), calculated as the number of non-synonymous variants per megabase (MB) of covered exome. **F** Clonality network plots derived from BCR sequencing data. Each circle represents a unique V(D)J sequence, and its size reflects the UMI-corrected read count. Clones which could be identified within an SHM tree are shown in red.

Since such in vitro studies are inherently limited, we next explored the feasibility of generating a systemic PDX in vivo model. After intravenous injection of unsorted leukemic peripheral blood mononuclear cells (PBMCs) collected at disease progression on ibrutinib into NOD.Cg-*Prkdc*^*scid*^ *Il2rg*^*tm1Wjl*^/SzJ (NSG) mice (Supplementary Methods), engraftment was observed. These F0 mice had to be sacrificed due to morbidity burden and enlarged spleens after a median of 138 days (range 78–167; Fig. 2A). Upon further intravenous passaging of tumor material isolated from spleens, median time to humane endpoint was <50 days in the F1 to F3 generation (Fig. 2A). Reflecting the systemic disease spread, all PDX mice showed infiltration of the spleen as assessed by immunohistochemistry (IHC) (Fig. 2B). While additional involvement of the bone marrow and/or liver was observed in most mice, leukemic disease was found in 25% (Fig. 2B). Importantly, the PDX mimics the patient lymphoma and the cell line in terms of morphology and immunophenotype (Fig. 1C,F**;** Fig. 2C). Using IHC and flow cytometry, we were able to confirm a consistent immunophenotype across spleen, bone marrow, peripheral blood and liver of the PDX model (Fig. 2C, D). In line with the molecular profiling of HaJo and the primary material, the genetic landscape and transcriptional signatures of the patient material were reflected by the PDX model across passages F0 to F3 (Fig. 2E and S2A). To characterize the clonal architecture, we further performed BCRseq of the refractory patient sample, the HaJo cell line, and longitudinal passages of the PDX model. A dominant clone representing over 90% of the unique molecular identifier (UMI)-corrected reads was identified, embedded within a network of SHM (Fig. 2F), which was highly consistent across all of the different conditions (Fig. S2,B).

Our patient case and recent literature clearly illustrates that the presence of *CCND1*-R in these rare, aggressive non-MCL lymphomas present a significant diagnostic and therapeutic challenge [7]. The conserved immunophenotype (CD19 + , BCL2 + , CD5-, CD10-, BCL6- and MUM1-) across patient material, HaJo cell line and corresponding PDX model shows striking similarities to the two previously reported transformed *CCND1*-R MZL cases [7]. Importantly, the genomic alterations identified at relapse, including trisomy 3 and deletions of *CDKN2A* and 8p and 18p, mirror changes recently described as characteristic of transformed SMZL [12]. This is further supported by our PCA analysis showing that HaJo clusters distinctly to MCL cell lines with an expression program more consistent with marginal zone/memory B-cell differentiation and NF-κB activation. The successful generation of a systemic, multi-organ PDX model with high, fast, and reproducible engraftment rates is a strength of our work. Unlike conventional cell line-derived xenografts or subcutaneous PDX models, commonly used in lymphoma research, our systemic model faithfully mimics systemic disease and therefore offers a unique opportunity to explore fundamental questions surrounding the role of *CCND1*-R in non-MCL lymphomas. Functional experiments using our models could elucidate the contribution of *CCND1*-R to tumor proliferation and engraftment, thereby determining its therapeutic relevance in addition to its diagnostic value. A further notable feature of our model is the observed loss of CD20 expression caused by biallelic *MS4A1* inactivation, a phenomenon relevant for understanding and potentially overcoming resistance to anti-CD20 directed immunotherapies [13]. Moreover, consistent overexpression of BCL2 in our model supports the rationale to explore BCL2 inhibition as a targeted therapeutic strategy.

Nonetheless, additional models representing the molecular heterogeneity of *CCND1*-R non-MCL are needed to comprehensively characterize this entity. These will be essential to differentiate between subtype-specific vulnerabilities and to refine diagnostic and therapeutic strategies.

Taken together, both the patient-derived HaJo cell line and corresponding systemic PDX in vivo model mimic key features of SOX11-negative *CCND1*-R non-MCL and thereby constitute valuable tools to study these rare but challenging lymphomas.

## Software

All statistical analyses and visualizations were conducted using R (version 4.3.2) or GraphPad Prism (version 10.4.2). Heatmaps and oncoprints were generated with ComplexHeatmap package (version 2.22.0) or pheatmap package (version 1.0.12).

## Supplementary information

Supplementary Methods and Supplementary Tables

Supplementary Figures

Supplementary Table 2

Supplementary Table 3

Supplementary Table 4

## Data Availability

The HaJo cell line and passages of the HaJo PDX model are available from the corresponding author upon reasonable request and pending institutional review board approval. Sequencing data generated in this study have been deposited in the European Genome-Phenome Archive (EGA) under controlled access in accordance with the Data Access Governance (DAG) protocol (WES: EGAS50000001432; WTS: EGAS50000001433; BCR-seq: EGAS50000001434).
