# Supplementary figures and images for: Establishment and characterization of a CCND1-rearranged non-mantle cell lymphoma cell line and patient-derived xenograft model
Source: Leukemia. 2026 Jan 26;40(3):666–70. doi: 10.1038/s41375-025-02849-3 (PMC12960241; doi:10.1038/s41375-025-02849-3)

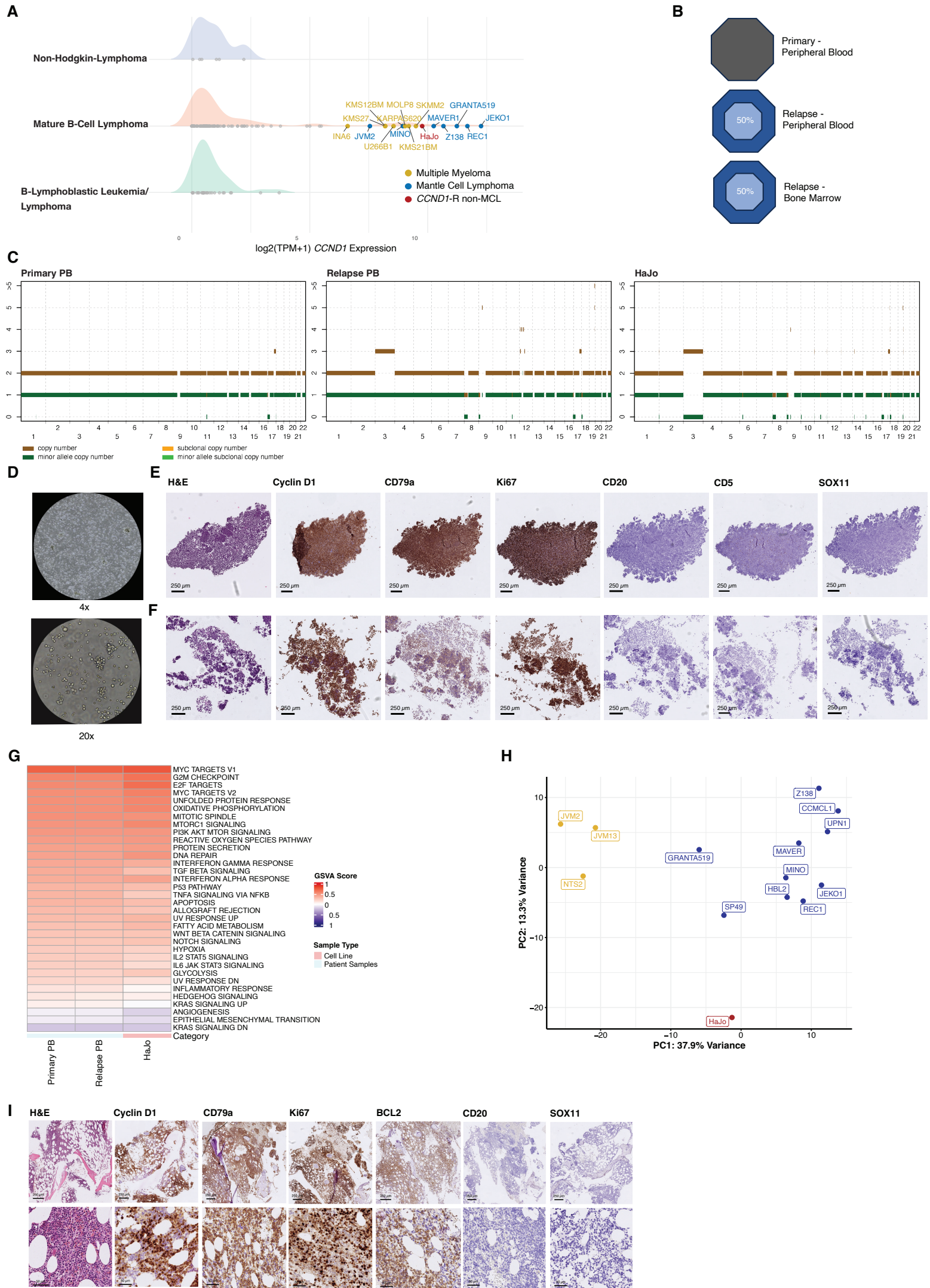

A

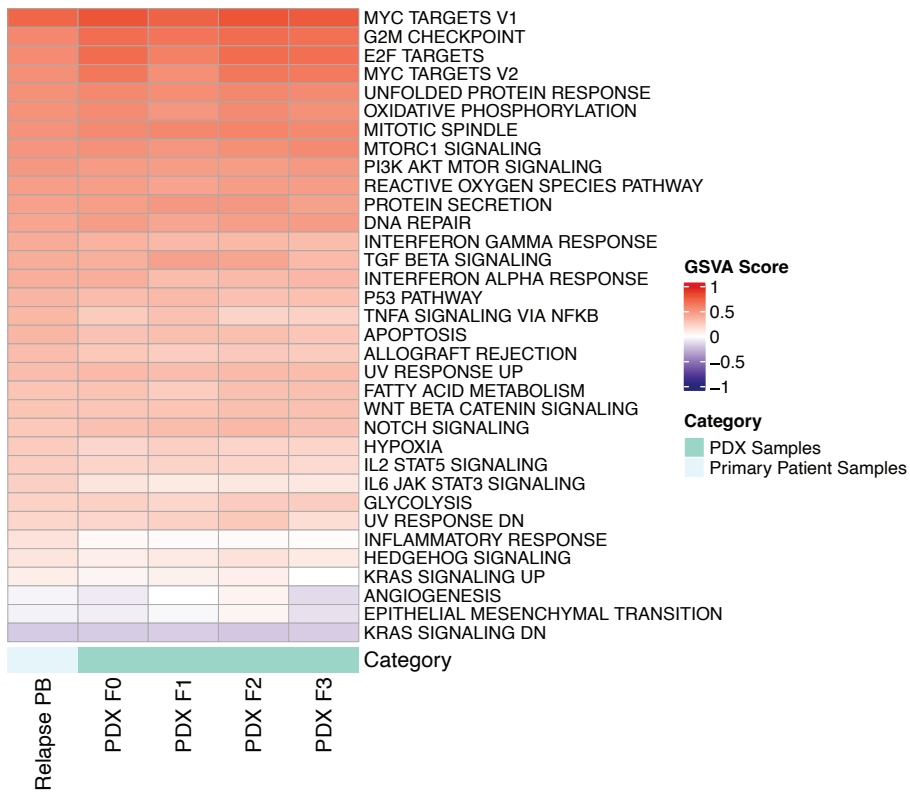

B

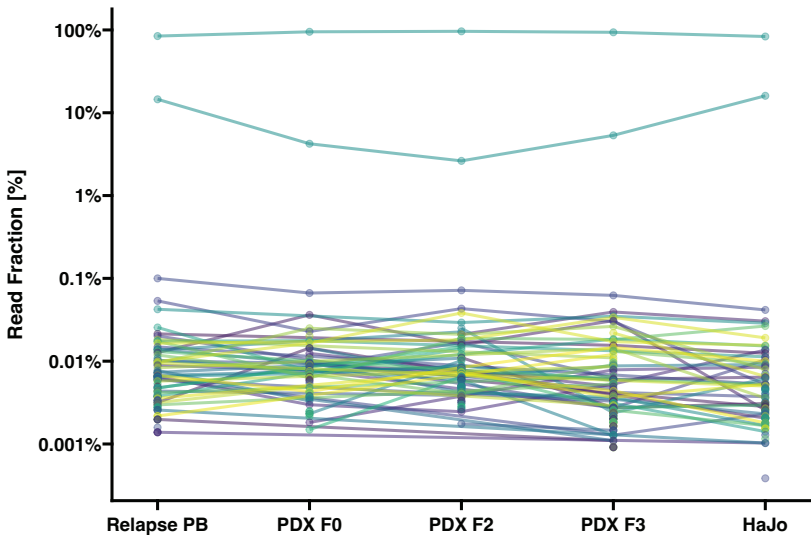

Supplement: Supplementary file 2 — Supplementary Figures [file 41375_2025_2849_MOESM2_ESM.pdf]
